# Q&A: What is the Open Connectome Project?

**DOI:** 10.1186/2042-1001-1-16

**Published:** 2011-11-18

**Authors:** Joshua T Vogelstein

**Affiliations:** 1 Department of Applied Mathematics and Statistics, John Hopkins University,100 Whitehead Hall, 3400 North Charles Street, Baltimore, MD 21218-2682, USA

## What is the connectome?

Although it has been over a century since neuroscientists first conjectured that networks of neurons comprise the brain, technology has limited high-throughput investigations of neural circuitry until very recently. In the last couple of decades, several experimental paradigms have arisen that are poised to finally begin studying neuroanatomy in a high-throughput fashion. In 2005, the term connectome was coined independently by Patric Hagmann [1] and Olaf Sporns [2], to describe the complete set of neural connections in a brain. Interestingly, both usages seemed to be referring to using Magnetic Resonance Imaging (MRI) to study human brain networks. Shortly thereafter, Narayanan "Bobby" Kasthuri and Jeff Lichtman published an article [3,4] suggesting that "connectome" should refer to connections between neurons, which one can infer using Electron Microscopy (EM) and fluorescence microscopy (e.g., brainbow animals [5]) "Projectome", they suggested, is more appropriate for MRI based studies. Yet, the word connectome stuck, and now refers to essentially any neuroscientific investigation of the relationship between (collections of) neurons, be they functional or structural.

## What are the technological advances that have enabled this project?

Perhaps the first paper describing the collection of high-throughput EM data in neuroscience was published by Winfried Denk in 2004, using serial block face EM to obtain large quantities of high-resolution images [6]. To capture such data quickly required the advent of high-speed cameras and large hard-drives. For example, the raw data from the Bock et al. [7] publication that we host required ~40 Terabytes (TB) of storage. 10 years ago it would have been prohibitively expensive to even store this data. Now, we have redundant copies of it, both in a two-dimensional image hierarchy and a custom volumetric database.

## What made you start the Open Connectome Project?

I was in Boston at the beginning of February 2011 talking to Clay Reid (of Harvard University) about some of our preliminary results on analysis of networks. His lab had just had a paper accepted in Nature, and I asked if the data could be made available on a central database which we could host. Fortunately for me, he had already been bitten by the open science bug, and had already distributed the data to Mark Ellisman of the Cell Centered Database. Thus, after getting approval from his collaborators, he agreed. The next day, we sent him 10 hard drives, his postdoc Wei-Chung Allen Lee uploaded the data and sent them back to us. Back at JHU, Eric Perlman, a graduate student in the Hopkins Storage Systems Lab run by Randal Burns, ordered a couple servers. I then called my friend Albert Cardona of the Institute of Neuroinformatics in Switzerland, knowing that he had developed CATMAID, a web app that could facilitate visualizing arbitrarily large 3D datasets. A few tweaks of their code from my brother and Eric, and a couple weeks later, the full high-resolution data was fully viewable online to anyone in the world. The Open Connectome Project (OCP) had launched!

## Did you say your brother?

Yup. I work with him on nearly everything I do. I love my work, and I love my brother. There are so many interesting questions and so many amazing people, my preference is to work with people I love on projects I love. I also work with my dad on some other top-secret stuff.

## OK, why open?

Science, in my view, is about continually improving our collective descriptions of how the universe works. We are so fortunate to live in a time and place that values and affords this pursuit sufficiently to enable large communities of people to essentially sit around and think all day to try to figure this stuff out. The laws of physics and chemistry and biology effect everyone on the planet equally, no matter his or her background, genetics, beliefs, etc. Why would we not want to share the beautiful data and explanations that science reveals with everybody? Also, though science operates within cultural and societal biases (like all human pursuits), perhaps it can be a stepping stone towards greater equality in the world.

Moreover, modern science is faced with complexities of dazzling proportions. Conceiving of even better explanations of our world vexes many of the great modern minds. Yet, only a vanishingly small proportion of people interested in these goals are poised to make significant contributions, often due merely to access. It seems counterproductive and downright silly to not at least allow anybody with sufficient interest to contribute by making our current datasets and inferences available to all.

## Why now?

The digital age dramatically changes the costs associated with dissemination of data in a number of ways. First, whether scientific instruments are counting photons or electrons or bosons, our tools for data acquisition are now largely electronic. Second, the internet facilitates inexpensive transmission of digital data. Our plan is to make the acquisition and dissemination of data both instantaneous and automatic. At the microscope, high-speed cameras will capture data at rates of 10TB per day, caught on local servers, and simultaneously piped to Hopkins where the data will immediately be ingested into a scientific database to enable instant access and analysis. Recently, some progressive scientists have begun sharing their data after publication. Unfortunately, publication is often delayed by years after data acquisition. Moreover, data sharing often takes significant activation energy. Because all the data will already be shipped via the internet and available to a small group of collaborators, the activation energy will now simply be flipping a switch to enable global access.

## Is connectomics particularly well suited to engage in open science?

Other fields have already embraced certain open science principles. For instance, physicists starting putting pre-publications on the arxiv over 20 years ago. More recently, astrophysicists have been amongst the first to realize that we share collective resources, and can therefore more efficiently capitalize on them by working together. Specifically, telescopes from around the world and solar system share their images, which means astrophysicists everywhere can work on understanding space, instead of simply trying to collect some data. Genetics is another example: it has become the norm to share sequence data.

One feature that connectomics shares with these other fields is that the data are so vast, that no human could possibly even look at all the data, much less analyse it. Currently, it takes about 1 day per human expert to manually trace a synapse from its source to its target. At that rate, it would take over 30 millions years for a person to merely see all the synapses once in a single human brain. Another property connectomics has is its infancy: because the field is so small, a panoply of low-hanging fruit remain wide open. And connectome science already actually has a rich history of open science. The connectome of a hermaphroditic C. elegans, a small nematode worm with only 302 neurons, was first published decades ago. Much of that data and more have been available on websites like http://www.wormbase.org and http://www.wormatlas.org for years.

## What services does the Open Connectome Project provide?

The first step in analyzing the EM connectome data is one of computer vision. Although our machines can currently store the data no problem, analyzing the data pushes beyond the limits of modern computer vision. So, we hope to facilitate "*alg-sourcing" *(a portmanteau of algorithm and outsourcing) to obtain collective annotations of the data. Storing the images and annotations in a graph and 3D space aware scientific database will facilitate efficient querying of the data to obtain novel scientific insights. We hope that the services we provide will be so useful to the data collectors that they will be sufficiently incentivized to enable us to host for them and share with the world.

## Once the data are annotated, how will they be used?

My main research area is actually on graph statistics, that is, we develop and apply tools for the statistical analysis of graphs. Our hope is to be able to utilize these tools on EM connectome data. There are myriad questions of interest to us, primarily, however, we take the following view: each brain-graph is a sample from some graph-valued random variable with unknown distribution. We'd love to be able to estimate that distribution. We'd also love to find which subgraphs (network motifs) are over represented relative to some null hypothesis about random graphs. Our list goes on and on. Another motivation for sharing the data is that we expect others to ask interesting neurobiological questions that we haven't even considered, and we want to know the answers to those questions too.

## Is the project dealing with any other kinds of connectomes?

Totally! We are scale agnostic. We believe that so much is unknown about the brain at many spatial and temporal resolutions, and so much potential utility is hidden in data sets with different resolutions, that we don't want to limit ourselves to any specific experimental approach. To that end, we have formed a collaboration with Michael Milham, founder of International Neuroimaging Data-sharing Initiative. Mike already has done so much for towards open connectomics, by running 1000 Functional Connectomes Project and the ADHD200 competition. Our plan with Mike is to build state-of-the-art scientific databases to store and process multimodal magnetic resonance imaging data. Thus, anybody will be able to upload their image data with one-click, and it will be incorporated into the database and processed automatically. People can then run analyses on their data, and compare with other datasets. As for the EM data, we hope these services will sufficiently incentive people to share their data (we recently described our intentions in a response to an NIH RFI).

In addition to our MR connectome efforts, we are in process of incorporating some light microscopy connectomes. Specifically, we are working with Logan Grosenick from Karl Deisseroth's lab to host their awesome light field imaging data from zebrafish and mice. And we are open to any other kind of data: if you've got massive connectome data, we want it.

## Are there other connectome projects?

There are several other connectome projects, which are all working in a collaborative rather than a competitive nature. The biggest project is the Human Connectome Project, which has received $40 million in NIH funding. This project will acquire and database MRI and other data from about 1200 subjects. In contrast, OCP does not collect its own data, rather, hosts whatever data wants to be shared. For example, we recently received a call from Scott Emmons, as he wanted to share both male and hermaphroditic *C. elegant *data that had been digitized and curated by David Hall. Other efforts like the Mouse Connectome Project, the Whole Brain Project, the Brain Observatory, and various efforts at the Allen Institute for Brain Science have nicely complementary goals.

## Where can I learn more?

**Figure 1:**
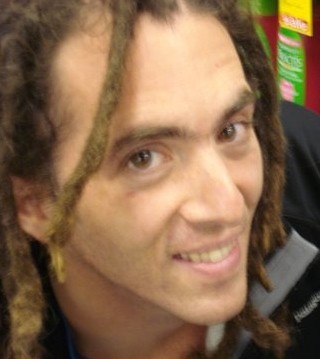
**Joshua T. Vogelstein Biography**. Joshua T. Vogelstein is an Assistant Research Scientist working with Professor Carey E. Priebe in the Department of Applied Mathematics and Statistics at Johns Hopkins University. After obtaining his bachelor's degree in Biomedical Engineering, he obtained a master's degree in Applied Mathematics & Statistics and a PhD in Neuroscience at the John Hopkins School of Medicine. The focus of his work is statistical connectomics: the art of connectome data collection, analysis and interpretation, with the aim of acquiring new insights into the human condition. He hopes to soon integrate the genome into his work so that he can study the shalome: the complete 'ome of an individual. The Open Connectome Project, which Joshua is helping to establish, aims to make state-of-the-art neuroscience available to anyone with computer access, regardless of their nature or nurture.

The OCP website has a bunch of links to technical articles, review articles, PR media, and related sites. Perhaps the best single source of information on open science available right now is Michael Nielson's book entitled, "Reinventing Discovery." To learn more about connectomes, I'm a fan of Olaf Sporns' book, "Networks of the Brain" or Sebastian Seung's upcoming book, "Connectome." Or just call/email me whenever. My email address is http://joshuav@jhu.edu, and my personal website is http://jovo.me/; I'm always happy to talk more about this stuff. A biography containing information about myself can be found in the legend to Figure 1.
